# Design and Evaluation of a Trunk–Limb Robotic Exoskeleton for Gait Rehabilitation in Cerebral Palsy

**DOI:** 10.3390/biomimetics11020101

**Published:** 2026-02-02

**Authors:** Hui Li, Ming Li, Ziwei Kang, Hongliu Yu

**Affiliations:** Institute of Intelligent Rehabilitation Engineering, University of Shanghai for Science and Technology, Shanghai 200093, China; lihui23825@usst.edu.cn (H.L.); 231320161@st.usst.edu.cn (M.L.); 233352493@st.usst.edu.cn (Z.K.)

**Keywords:** cerebral palsy, gait rehabilitation, trunk-limb exoskeleton, self-aligning mechanism, multi-joint coordination

## Abstract

Most pediatric exoskeletons for cerebral palsy (CP) focus on lower-limb assistance and neglect trunk control, limiting rehabilitation outcomes. This study presents a self-aligning trunk–limb exoskeleton that integrates trunk stabilization with active lower-limb support. The design includes a hip–waist rapid adjustment mechanism, a bioinspired gear-rolling knee joint, modular thigh–shank structures, a trunk support module, and a body-weight support device. To enable transparent and coordinated assistance under pathological gait conditions, a continuous gait progress-based multi-joint control framework is developed. Joint motion is described as continuous gait progress over the full gait cycle (0–100%), and joint-specific progress estimates are fused into a unified system-level reference using observability-weighted circular statistics. Inter-joint coordination is achieved through phase-consistency-based temporal modulation implemented, enabling smooth synchronization while preserving joint-level autonomy and motion continuity. Technical evaluation—comprising kinematic misalignment analysis, simulation validation, and gait trials—demonstrated a 66.8% reduction in hip misalignment and an 87.4% reduction in knee misalignment. Gait parameters under exoskeleton-assisted walking closely matched baseline walking, confirming natural kinematic preservation without interference. These results indicate that the proposed trunk–limb exoskeleton improves human–robot synergy, enhances postural stability, and provides a promising solution for pediatric gait rehabilitation in CP.

## 1. Introduction

Cerebral palsy (CP) is a non-progressive developmental brain injury disease characterized by permanent motor impairments, particularly affecting gait and posture control [1,2]. Independent walking is considered a primary functional goal for individuals with CP [3]. Over the past few decades, wearable robotic exoskeletons have emerged as a valuable tool, enabling individuals to walk freely in diverse settings, both indoors and outdoors [4]. These devices offer a more realistic, adaptable, and engaging training environment and provide a promising means for CP children to correct abnormal walking patterns and enhance independent ambulation [5].

According to existing research, the goal of robotic exoskeleton assistive gait rehabilitation for CP children focuses on gait correction [5]. Ambulation dysfunction results from the interaction between lower-limb joint motion and trunk motion. Abnormal torso posture is another significant feature in children with CP and impairs gait function through walking balance, which further affects head correctly integrating vestibular and visual information required for balance-related functions [6]. When lower-limb gait is impaired, compensatory motion strategies of trunk are always generated, which may lead to secondary musculoskeletal defects under long-term repetition. Therefore, torso-level intervention is necessary for effective learning of correct walking patterns.

At present, research on incorporating trunk assist mechanisms into the exoskeleton system remains limited, and studies that jointly address gait and trunk activity to improve human walking mechanisms in CP children are largely absent. The only reported research on wearable exoskeleton related to gait improvement and torso intervention is the CPwalker, which provides acoustic feedback to guide user self-adjustment based on body orientation estimated from two IMU sensors placed on the chest and head [7]. Although this strategy partially enhances cognitive interaction between the child and the robot, it does not achieve direct gait correction or effective postural control. An effective device must not overlook these critical features. In this regard, there is a strong possibility that the development of trunk–limb exoskeleton is a promising breakthrough point for the gait rehabilitation in children with CP.

From a design perspective of trunk–limb exoskeleton, the challenges associated with CP extend beyond merely addressing the discomfort arising from misalignment of exoskeleton and biological axes of rotation [8], they also encompass the necessity to mechanically accommodate the physiological characteristics of pediatric users. Children with CP exhibit various musculoskeletal disabilities and diverse gait patterns, including but not limited to crossing leg, scissor gait, and crouch gait. Consequently, the exoskeleton must have a wide range of adaptability to accommodate variations in limb anatomies, movement patterns, and posture states, while maintaining a simplified mechanical layout to facilitate structural adjustment and configuration.

To date, many studies have focused on adaptive exoskeleton mechanisms to improve kinematic compatibility for pediatric populations with CP [8]. Introducing redundant joints has been a popular research method [9]. For instance, Beil and Junius K. et al. incorporated multiple hinges and sliders into each rotational DOF [10,11,12,13,14,15]. Celebi [12] and Lee [13] decoupled rotational and translational motions to achieve improved kinematic matching. Other approaches employ biomimetic designs, such as curved rails [16], cams [17], gears [18], and linkages [19,20], to replicate joint motion paths. More recent work has introduced remote-center-of-rotation mechanisms to follow the knee’s instantaneous center of rotation (ICR). However, a major limitation of these mechanisms is the inevitable increase in system mass and mechanical complexity, which hinders practical implementation and compromises user comfort.

Achieving effective assistance in pediatric CP populations remains challenging due to the high variability and irregularity of pathological gait [21]. Compared with healthy adult gait, CP gait often exhibits inconsistent step timing, reduced joint excursions, and asymmetric inter-joint coordination, which substantially complicates real-time exoskeleton control. Inappropriate or mistimed assistance can not only degrade walking performance but also interfere with residual voluntary control, underscoring the need for transparent control strategies that respect the user’s natural movement intent [22]. Transparent control aims to provide intuitive and unobtrusive assistance, allowing the child to remain the primary driver of motion while the exoskeleton delivers timely support—an aspect particularly critical for pediatric users, who rely heavily on sensory feedback for motor learning. However, many existing approaches based on single-joint gait phase estimation, predefined finite-state machines, or fixed timing references derived from healthy gait patterns often fail to accommodate the multi-joint phase inconsistency inherent in CP gait. In particular, deriving a global timing reference from a single joint may lead to unsafe assistance when the joint exhibits spasticity, limited range of motion, or irregular activation.

This paper presents a trunk–limb exoskeleton designed to improve gait function in children with CP. The system integrates trunk assistance as an explicit postural constraint during overground walking and emphasizes kinematic synergy between the human body and the exoskeleton. We also propose a unified gait phase-based adaptive multi-joint control method that provides a common temporal reference for coordinated assistance. Local gait phases are independently estimated at multiple joints and fused into a system-level reference phase, while a phase-consistency-based coupling strategy gradually aligns joint-level control. Adaptive regulation of fusion weights and coupling gains enhances robustness to pathological gait variability while preserving joint-level motion characteristics, enabling transparent, intuitive, and stable assistance for CP rehabilitation.

## 2. Materials and Methods

### 2.1. Design of a Self-Aligning Trunk–Limb Exoskeleton

**Figure 1:**
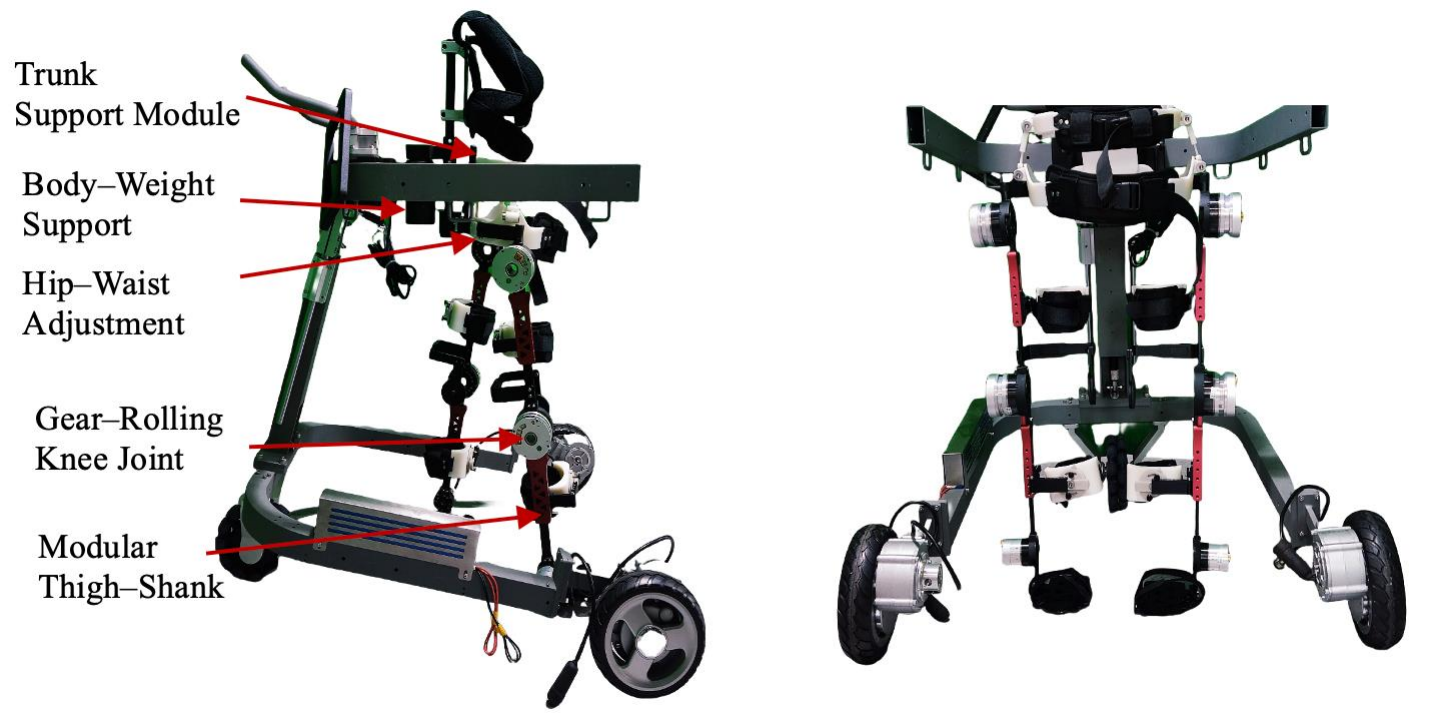
The structure of trunk–lower-limb exoskeleton.

During ambulation, coordinated interactions between the torso and lower limbs are essential for maintaining whole-body stability and balance [23,24,25]. Figure 1 illustrates the overall design concept of the proposed system. The exoskeleton consists of a trunk support module that enhances dynamic torso stability and a lower-limb module that assists leg flexion-extension motions. This section focuses on the configuration design of the integrated trunk–limb exoskeleton.

#### 2.1.1. Non-Anthropomorphic Hip–Waist

**Figure 2:**
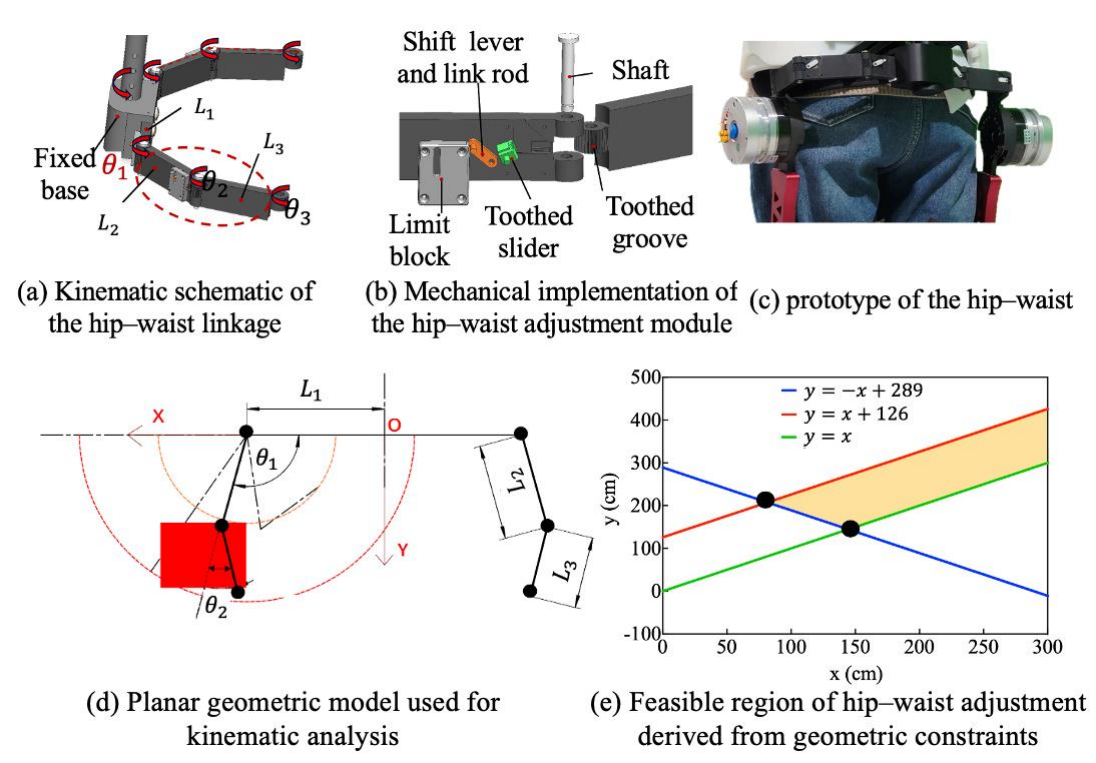
Hip–waist rapid adjustment mechanism.

To enable unobstructed hip motion within the exoskeleton framework, our design employs a self-aligning adjustment chain composed of multiple revolute joints to ensure proper fit and alignment. As shown in Figure 2, the kinematic chain of internal/external rotation is composed of three revolute joints ($\theta_{1}$, $\theta_{2}$, and $\theta_{3}$) and three rigid links ($L_{1}$, $L_{2}$, and $L_{3}$), which conform to the external profile of the human waist. Through carefully selecting link lengths, the mechanism covers the required adjustment area while simplifying the donning process. During wearing, the exoskeleton can be easily aligned and attached to the lateral side of the thigh by adjusting only the distal link, thereby reducing the setup time and improving usability.

A Cartesian coordinate system is established, with the origin *O* defined as the intersection of the exoskeleton central axis and the waist connector, and the *x*-axis aligned with the waist connector. Let $L_{1}$ denote the minimum lateral distance from the hip joint edge to the central axis, i.e., half of the minimum body width. The lengths of the other two links are denoted by $L_{2}$ and $L_{3}$, with joint angles $\theta_{1}$ and $\theta_{2}$, respectively. The distal endpoint can be expressed as:

(1) $$\left\{\begin{array}{c} p_{x}=L_{1}-L_{2}\cos\theta_{1}-L_{3}\cos(\theta_{1}-\theta_{2})\\ p_{y}=L_{2}\sin\theta_{1}+L_{3}\sin{(\theta }_{1}-\theta_{2}) \end{array}\right..$$

The feasible coordinate range of hip satisfy:

(2) $$95\leq p_{x}\leq 155,$$

(3) $$120\leq p_{y}\leq 260.$$

The reachable workspace of endpoint hip corresponds to an annular region bounded by two concentric circular boundaries:

Inner circle:

(4) $${\left(x-L_{1}\right)}^{2}+y^{2}={\left(L_{2}-L_{3}\right)}^{2}.$$

Outer circle:

(5) $${\left(x-L_{1}\right)}^{2}+y^{2}={\left(L_{2}+L_{3}\right)}^{2}.$$

For the linkage design to be feasible, the annular workspace must encompass the target area. This condition imposes the following constraints on the linkage lengths:

(6) $$\left\{\begin{array}{c} L_{2}+L_{3}>289\\ 0<L_{2}-L_{3}<126 \end{array}\right.$$

By solving these constraints, the feasible design region for the link lengths $\left(L_{2},L_{3}\right)$ is obtained, as illustrated by the shaded region in Figure 2e. The final link lengths and joint motion ranges were selected to ensure that the annular workspace fully encompassed the required adjustment region. This guarantees sufficient adaptability for users with different body sizes. During donning, only the distal link requires adjustment, enabling rapid side attachment of the exoskeleton to the thigh surface and thereby reducing wearing time.

In addition, a joint-locking mechanism was integrated at the articulation interfaces between link-link and link-frame components. This design allows selective constraints of the system’s DOFs. The flexible locking architecture converts these joints into rigid linkage structures to adapt to the user’s dynamic movements. To achieve this mode-specific joint behavior, a locking unit consists of a toothed slider, a toothed groove, a link rod, a shift lever, and a limit block. The toothed slider and toothed groove are affixed to adjacent links. Mechanical locking is realized via engagement between their matching tooth profiles. As shown in Figure 2b, the shift lever moves vertically within the sliding slot of the limit block, transmitting displacement through the connected link rod to laterally drive the toothed slider. When the slider reaches its terminal position, it engages with the toothed groove at the joint, forming a locked configuration. To prevent unintentional disengagement during locomotion due to vibration or external forces, a dead-point design principle is implemented. In the locked state, the resultant force acting on the slider is orthogonal to its displacement direction, thereby ensuring mechanical stability. Unlocking is achieved by upward movement of the shift lever to disengage the toothed interface.

#### 2.1.2. Anthropomorphic Knee Joint

**Figure 3:**
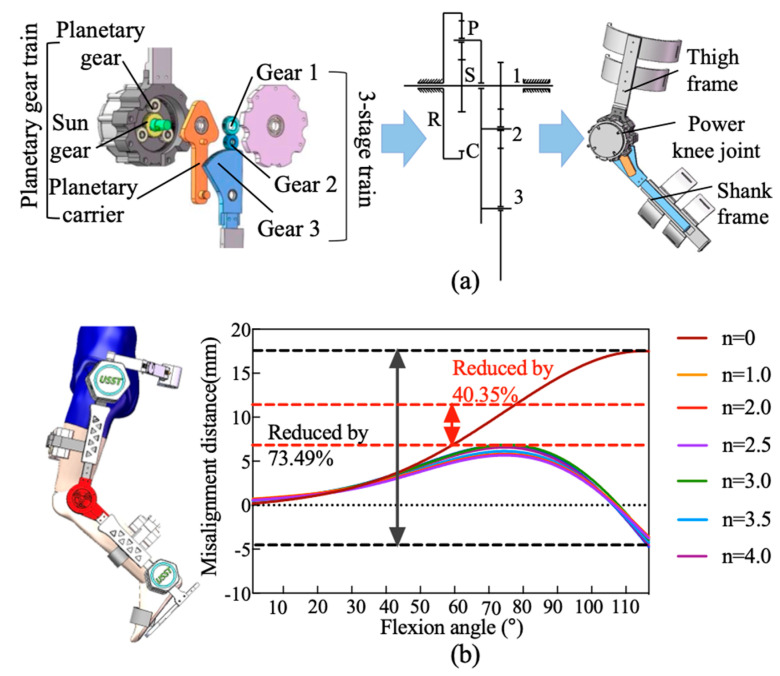
The design of bioinspired gear-rolling knee joint. (**a**) Planetary gear train. (**b**) Flexion angle.

The biological knee exhibits combined rotational and translational motions [24,25]. A bioinspired mechanical knee joint is implemented using a gear-based transmission mechanism. As shown in Figure 3, a planetary gear train is employed to emulate femoral-tibial rolling kinematics, which reproduces the rotation angular, and a three-stage transmission mechanism is employed to dynamically adjust the gear meshing positions, compensating for ICR misalignment.

The planetary gear system consists of a sun gear (s), a planet gear (p), a ring gear (r), and a planet carrier (c). In this configuration, the ring gear (r) is rigidly fixed to the thigh frame, while the sun gear (s) is coupled to the motor output shaft and serves as the driving gear. The rotational motion of the planet carrier (c) and its accompanying planet gear (p) emulates the relative rolling motion of the tibia about the femur during flexion–extension cycles. The knee rotation angle (α) is determined by integrating the angular velocity of the planet carrier ($\omega_{c}$) over time:

(7) $$\alpha =\int \omega_{c}dt,$$

where $\omega_{c}$ is derived from the sun gear’s angular velocity ($\omega_{s}$) and the gear ratio:

(8) $$\omega_{c}=\left(\frac{z_{s}}{z_{b}+z_{s}}\right)\cdot \omega_{s}.$$

In (7) and (8), $\omega_{s}$ represents angular velocity of the sun gear (governed by motor input). $z_{s}$ and $z_{r}$ denote the teeth count on the sun gear and the ring gear, respectively.

To compensate for ICR misalignment, the three-stage gear transmission is mounted on the planetary carrier, located on the side opposite to the planetary gear assembly. The transmission system consists of a planet carrier and three external gears (gear 1, 2, and 3) that mesh with each other. The input stage gear 1 is connected to sun gear and directly driven by the motor. The output stage gear 3 has a portion of its tooth profile engaged to the lower leg connecting rod. Gear 2 serves as an idler, reversing the rotational direction of gear 3 to ensure proper kinematic alignment. By dynamically adjusting gear meshing positions, this three-stage transmission system with a controlled rolling mechanism effectively substitutes the sliding motion between the tibia and the femur. The rotational angle *β* could be expressed as the time integral of the angular velocity of gear 3 ($\omega_{3})$:

(9) $$\beta =\int \omega_{3}dt$$

(10) $$\omega_{3}=\frac{z_{2}}{z_{3}}\cdot \omega_{2}$$

where $\omega_{2}$ denotes the rotational speed of gear 2; $z_{2}$ and $z_{3}$ represent the teeth counts of gears 2 and 3, respectively.

Gear 1 and sun gear are both mounted on the input shaft, resulting in identical angular velocities. The rotational speed of gear 2 is influenced by two opposing motions from the meshing transmission of gear 1 and the rotation of the planet arm. Defining the direction of rotation of the sun gear as positive, the resultant velocity is given by:

(11) $$\omega_{2}=\omega_{R2}-\omega_{12}=\frac{z_{1}}{z_{2}}\cdot \omega_{C}-\frac{z_{1}}{z_{2}}\cdot \omega_{1}=\frac{z_{1}}{z_{2}}\cdot \left(\omega_{C}-\omega_{1}\right)$$

where $\omega_{R2}$ represents the angle change in gear 2 due to the revolution of planet carrier, $\omega_{12}$ arises from gear 1’s meshing action. Additionally, $z_{1}$ and $z_{2}$ denote the teeth counts of gear 2 and Gear 3, respectively. Once the gear teeth counts are determined, the angles α and β maintain a fixed instantaneous speed ratio. Therefore, this ratio could be expressed as:

(12) $$n=\frac{\alpha }{\beta }=\frac{\omega_{c}}{\omega_{3}}=\frac{z_{s}\cdot z_{3}}{z_{r}\cdot z_{1}}$$

To determine the optimal gear parameters, a computational model of the human knee joint and the exoskeleton was developed in MATLAB R2022b (MathWorks, Natick, MA, USA) using the Simscape module. In this model, the biological knee joint was represented by the femur and tibia, with its kinematic behavior described using established relationship among rolling distance, sliding distance, and flexion angle, as reported by Kok Meng Lee [26]. For the exoskeleton component, biomimetic performance was evaluated based on the relative displacement of the shank linkage along the anatomical trajectory of the human leg during flexion–extension. By comparing this displacement with the physiological knee motion, the degree of kinematic similarity between the exoskeleton and the human joint could be quantitatively assessed. The finalized gear parameters, derived from this modeling and evaluation process, are summarized in Table 1.

#### 2.1.3. Mechanical Leg for Morphological Adaptation

**Figure 4:**
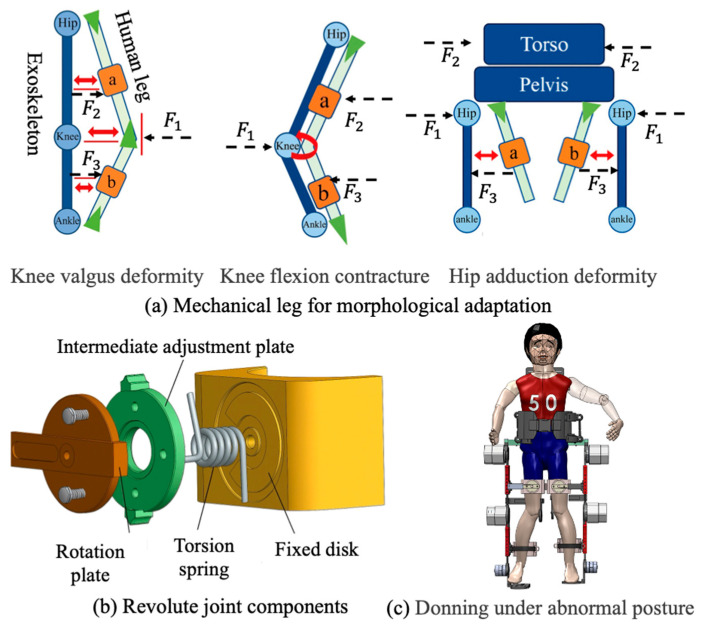
The structure of exoskeleton thigh and shank.

The exoskeleton’s lower-limb mechanical structure is modularly divided into thigh and shank segments. To address common lower-limb deformities in pediatric cerebral palsy patients—such as genu varum and genu valgum—each leg segment integrates adjustable modules at the human–machine interface that enable alignment with individual anatomical variations. As shown in Figure 4, prismatic and revolute joints were embedded into the leg brace modules to allow spatial repositioning and angular correction. The prismatic joint is implemented using a dual-link rail-slider assembly, allowing horizontal translation between the brace and the mainframe. The revolute joint consists of a wave spring, torsion spring, intermediate adjustment disk, fixed disk, rotation shaft, and rotation disk, allowing variation in the rotation range with limb posture to arrive to morphological adaptation.

Angular adjustment is realized via coordinated interaction among the fixed disk, intermediate adjustment disk, and rotation disk. The fixed disk includes preset angular limit slots that constrain the rotation range of the adjustment disk to −30° to +20°, covering the expected spectrum of limb malalignments. During fitting, the leg brace modules are manually adjusted to appropriate offsets, and locking mechanisms are used to fix both translational and rotational.

#### 2.1.4. Trunk Exoskeleton for Upright Support

Trunk stability is the core of good gait that responsible for maintaining the body upright, adjusting the changes in gravity center and performing stable walking movements [27,28,29]. A trunk upright-support module composed of a thoracolumbar spine exoskeleton was designed to help users keep a correct upright position during walking. It can be removed, adjusted, or retained to provide different levels of support for users’ actual torso control ability.

**Figure 5:**
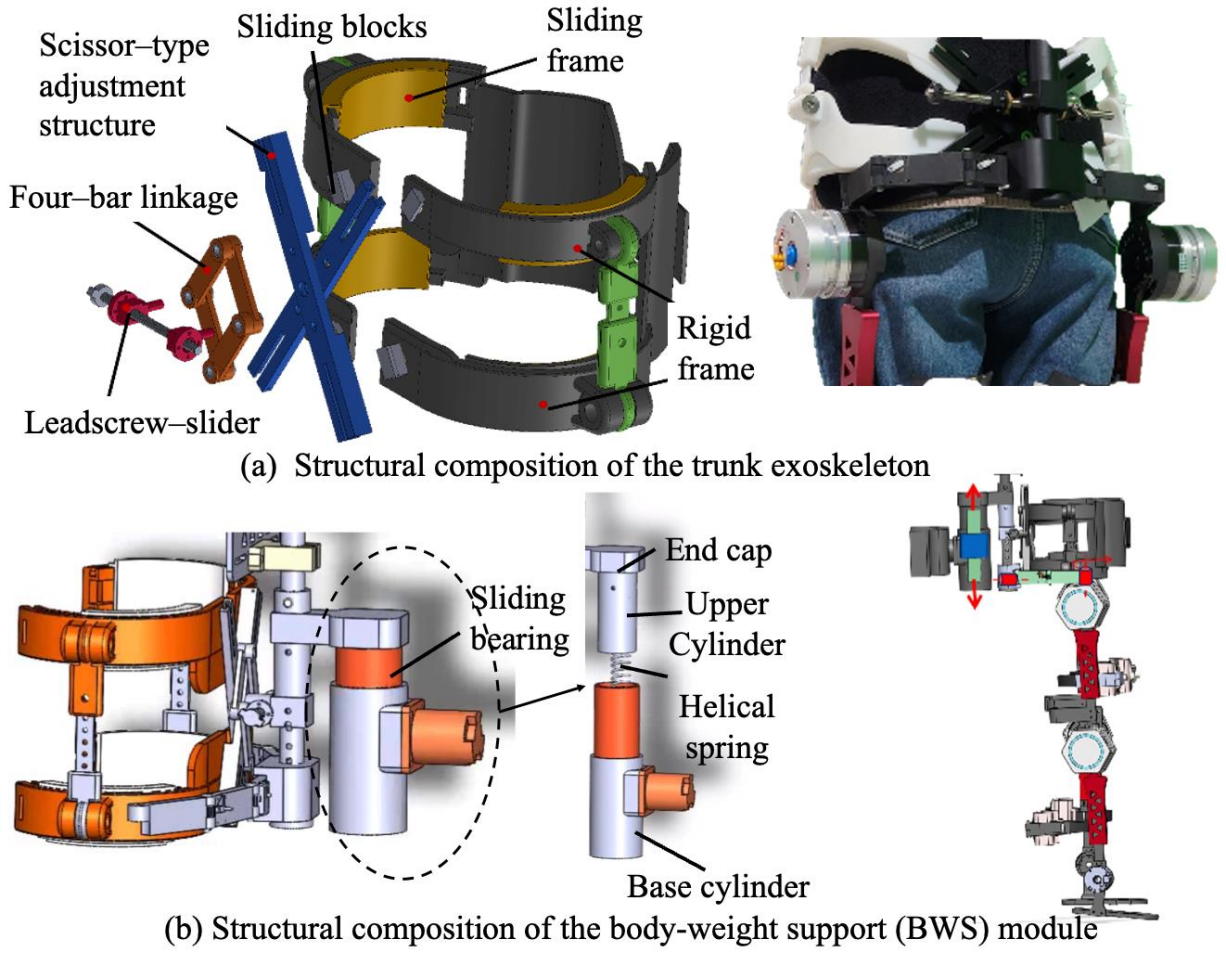
The structure of trunk exoskeleton and body-weight support component.

As shown in Figure 5a, the thoracolumbar assistance module consists of a rigid trunk fixation frame, a rapid body-size adjustment mechanism, and a trunk posture adaptation mechanism. The rigid frame is divided into upper and lower groups, attached respectively to the chest and waist, and connected to a scissor-type adjustment structure through sliding blocks; by adjusting the sliding displacement and opening angle, the device can accommodate different body sizes. To improve structural rigidity and prevent deformation under load, a leadscrew–slider assembly was integrated with the scissor structure by four-bar linkage, where the self-locking property of the leadscrew ensures stability after adjustment. Additional waist straps are applied to increase intra-abdominal pressure and further stabilize trunk posture. To allow adaptation to gait-induced trunk motion, the rigid frames composed of sliding rails and sliders permit ${-100}^{{}^{\circ}}$ to ${+100}^{{}^{\circ}}$ rotational compensation, together with a lateral revolute pair that accommodates trunk tilt within ${-80}^{{}^{\circ}}$ to ${+80}^{{}^{\circ}}$, thereby satisfying the requirements of natural walking.

Although the trunk module is passively supported, its assistance capacity is adjustable through structural configuration. The lateral width of the trunk frame is adjustable to accommodate different body sizes, while the assisted trunk segment can be repositioned vertically along the torso. For children with severe cerebral palsy and extremely limited active trunk control, the trunk support can be adjusted to the thoracic level to provide higher-level upper-body stabilization. For users with relatively preserved motor control, the assistance segment can be positioned below the waist, offering partial support while allowing greater trunk mobility. This configuration enables scalable trunk assistance across a wide range of functional abilities.

#### 2.1.5. Body-Weight Support Module

Children with CP often exhibit impaired balance and limited control ability. A body-weight support (BWS) device, also referred to as an unloading training device, was integrated into the system to reduce the load on the lower limbs during walking. In the present design, as shown in Figure 5b, the BWS device was mounted between the powered walker and the exoskeleton, and consists of a sliding bearing, base cylinder, helical spring, movable end cap, upper cylinder, and elastic pad. The upper and lower end caps are fixed to the exoskeleton via pin joints, while the upper cylinder is connected to the movable end cap using bolts.

During gait, vertical displacements of the exoskeleton are transmitted to the helical spring via the end caps. When the user’s center of mass shifts downward, the spring undergoes compression to store mechanical energy; conversely, when the center of mass moves upward, the stored energy is released to assist vertical motion and provide additional upward thrust. The base cylinder was securely attached to the walker’s frame by bolts, with vent holes incorporated at its lower surface to prevent undesired pneumatic resistance caused by air compression. Furthermore, sliding bearings were integrated between the upper and base cylinders to minimize friction during relative displacement, thereby improving energy efficiency and enhancing user comfort.

### 2.2. Control Strategy

Children with CP commonly exhibit abnormal inter-joint timing, reduced joint excursion, and irregular stride-to-stride variability, which challenge gait phase estimation and multi-joint coordination in lower-limb exoskeletons. Conventional finite state-based methods discretize walking into predefined states and rely on event detection or single-joint timing, which may become unreliable under pathological gait conditions.

**Figure 6:**
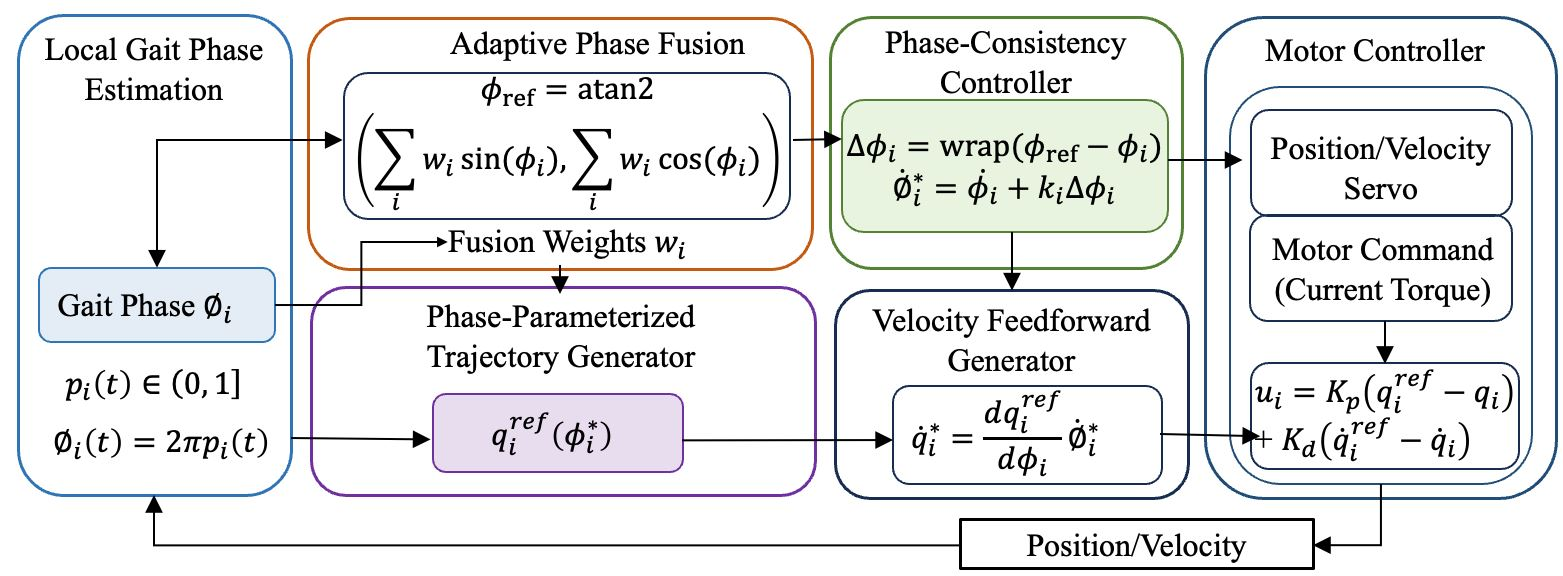
Unified gait phase-based adaptive multi-joint control and motor-level implementation.

In this study, we propose a continuous gait progress-based control framework that represents joint motion as a percentage of the entire gait cycle (0–100%), rather than as discrete gait states. Multiple joint-specific gait progress estimates are unified into a system-level reference using observability-weighted circular statistics, yielding a robust global timing reference without explicit phase classification. The adaptive realization of the unified gait phase across different joints is illustrated in Figure 6.

#### 2.2.1. Joint Continuous Gait Progress Estimation

For each actuated joint $i\in \left\{1,2,3\right\}$, motor-side sensing provides joint angle $q_{i}\left(t\right)$ and angular velocity $\dot{q_{i}}\left(t\right)$. We define continuous gait progress $p_{i}\left(t\right)\in \left(0\right.,\left.1\right]$, which represents the joint’s progression through the current gait cycle. For circular operations, gait progress is mapped to an angular phase

(13) $$\emptyset_{i}\left(t\right)=2\pi p_{i}\left(t\right)\in \left(0\right.,\left.2\pi \right].$$

This formulation treats gait timing as a circular variable, where 0% and 100% correspond to the same gait event.

The proposed method does not classify gait into discrete phases using a finite-state machine. Instead, each joint estimates its continuous gait progress by referencing a nominal joint trajectory defined over the entire gait cycle.

For each actuated joint i, an offline nominal profile $q_{i}^{nom}\left(\emptyset \right)$ ($\emptyset_{i}\in \left(0\right.,\left.2\pi \right]$), is constructed from calibration walking data. During online operation, the instantaneous phase estimate is obtained by solving

(14) $${\hat{\emptyset }}_{i}\left(t\right)=\arg min{\left(q_{i}\left(t\right)-q_{i}^{nom}\left(\emptyset \right)\right)}^{2},$$

subject to a continuity constraint:

(15) $${\hat{\emptyset }}_{i}\left(t\right)\leftarrow unwrap\left({\hat{\emptyset }}_{i}\left(t\right),{\hat{\emptyset }}_{i}\left(t-∆t\right)\right),\;{\hat{\emptyset }}_{i}\left(t\right)\geq 0.$$

Here, unwrap(·) selects the phase equivalent (mod 2π) closest to the previous estimate, ensuring monotonic evolution and preventing phase regression. The resulting gait progress is

(16) $$p_{i}\left(t\right)=\frac{{\hat{\emptyset }}_{i}\left(t\right)}{2\pi }.$$

#### 2.2.2. Joint-Level Observability and Adaptive Weighting

Because joint motion reliability may vary due to spasticity or limited excursion, a joint-level observability index $\eta_{i}\in \left(0\right.,\left.1\right]$ is introduced to quantify the reliability of each joint’s gait progress estimate. The observability index is computed from bounded measures of joint motion amplitude and signal stability and is constrained by design.

Adaptive fusion weights are defined as

(17) $$w_{i}\left(t\right)=\frac{\eta_{i}\left(t\right)+\varepsilon }{\sum_{j=1}^{N}\eta_{j}\left(t\right)+\varepsilon },\;\;\;\;\;\;w_{i}\geq 0,\;\;\;\;\;\;\sum_{i}w_{i}=1,$$

where ε>0 ensures numerical robustness.

Each joint phase is embedded on the unit circle:

(18) $$\nu_{i}\left(t\right)=\left[\begin{array}{c} cos\phi_{i}(t)\\ sin\phi_{i}(t) \end{array}\right].$$

The weighted circular mean vector is computed as

(19) $$V\left(t\right)=\sum_{i=1}^{N}w_{i}\left(t\right)\nu_{i}\left(t\right).$$

The unified reference gait phase is then defined by

(20) $$\phi_{ref}=atan2\left(\sum_{i}w_{i}sin\phi_{i}\left(t\right),\sum_{i}w_{i}cos\phi_{i}\left(t\right)\right),$$

and converted to a unified gait progress

(21) $$p_{ref}\left(t\right)=\frac{\phi_{ref}\left(t\right)}{2\pi }\in \left(0\right.,\left.1\right].$$

This formulation preserves the circular topology of gait timing and avoids boundary artifacts near 0–100%.

#### 2.2.3. Phase-Consistency Coupling for Multi-Joint Coordination

The circular deviation between joint and reference progress is defined as

(22) $$∆\phi_{i}\left(t\right)=wrap(\phi_{ref}\left(t\right)-\phi_{i}\left(t\right)),$$

where wrap(·) enforces circular boundedness.

Let ${\dot{\phi }}_{i}\left(t\right)$ denote the nominal phase rate derived from $\phi_{i}\left(t\right)$. The corrected phase rate is

(23) $${\dot{\emptyset }}_{i}^{*}\left(t\right)=\dot{\phi_{i}}\left(t\right)+k_{i}\left(t\right)∆\phi_{i}\left(t\right),$$

where $k_{i}\left(t\right)>0$ is a bounded coupling gain. This law adjusts the trajectory progression rate rather than imposing instantaneous kinematic constraints, enabling gradual synchronization across joints.

Coupling gains are scheduled within predefined bounds:

(24) $$k_{i}\left(t\right)\in \left[k_{i}^{max},\;k_{i}^{min}\right],$$

and may vary with gait progress to balance synchronization and transparency.

The corrected phase $\emptyset_{i}^{*}$ is obtained by integrating ${\dot{\emptyset }}_{i}^{*}$ and is used to parameterize joint references:

(25) $$q_{i}^{ref}\left(t\right)=q_{i}^{ref}\left(\phi_{i}^{*}\left(t\right)\right).$$

The corresponding reference joint velocity is given by

(26) $${\dot{q}}_{i}^{*}\left(t\right)=\frac{dq_{i}^{ref}}{d\phi_{i}}{\dot{\emptyset }}_{i}^{*}\left(t\right).$$

Motor commands are generated using a low-level servo with velocity feedforward:

(27) $$u_{i}=K_{p}\left(q_{i}^{ref}-q_{i}\right)+K_{d}\left({\dot{q}}_{i}^{ref}-{\dot{q}}_{i}\right),$$

where $u_{i}$ denotes the motor torque command, and $K_{p}$ and $K_{d}$ are servo gains. In this structure, multi-joint coordination is realized through smooth modulation of trajectory timing rather than direct enforcement of joint motion.

In this formulation, phase consistency influences motor behavior exclusively through the feedforward velocity term, allowing the servo controller to gently accelerate or decelerate joint motion in accordance with the corrected phase evolution. When the local joint phase aligns with the reference phase (i.e., $∆\phi_{i}=0$), the feedforward term reduces to the nominal trajectory velocity, and the controller behaves as a conventional joint servo without additional intervention.

This motor-level realization ensures continuity and boundedness of control commands, while preserving joint-level autonomy and responsiveness. As a result, inter-joint coordination is achieved via temporal synchronization rather than rigid kinematic constraints, which is essential for transparent assistance in CP exoskeleton applications.

## 3. Results

In this section, a technical validation of the proposed self-aligning exoskeleton mechanism is discussed, with the aim of establishing a robust benchmark for future clinical applications involving CP patients. All experimental procedures were conducted on five healthy participants (*N* = 5; age 25.4 ± 2.1 years). Prior to participation, each subject was fully informed of the study protocol and provided written informed consent. The experimental protocol was reviewed and approved by the Ethics Committee of Xinhua Hospital affiliated with Shanghai Jiao Tong University School of Medicine (Approval No. XHEC-C-2024-202-1). All quantitative results reported in this section are based on a limited sample size (*N* = 5) and are intended to illustrate engineering feasibility and consistent trends rather than inferential statistical conclusions.

### 3.1. Misalignment Evaluation and Results

**Figure 7:**
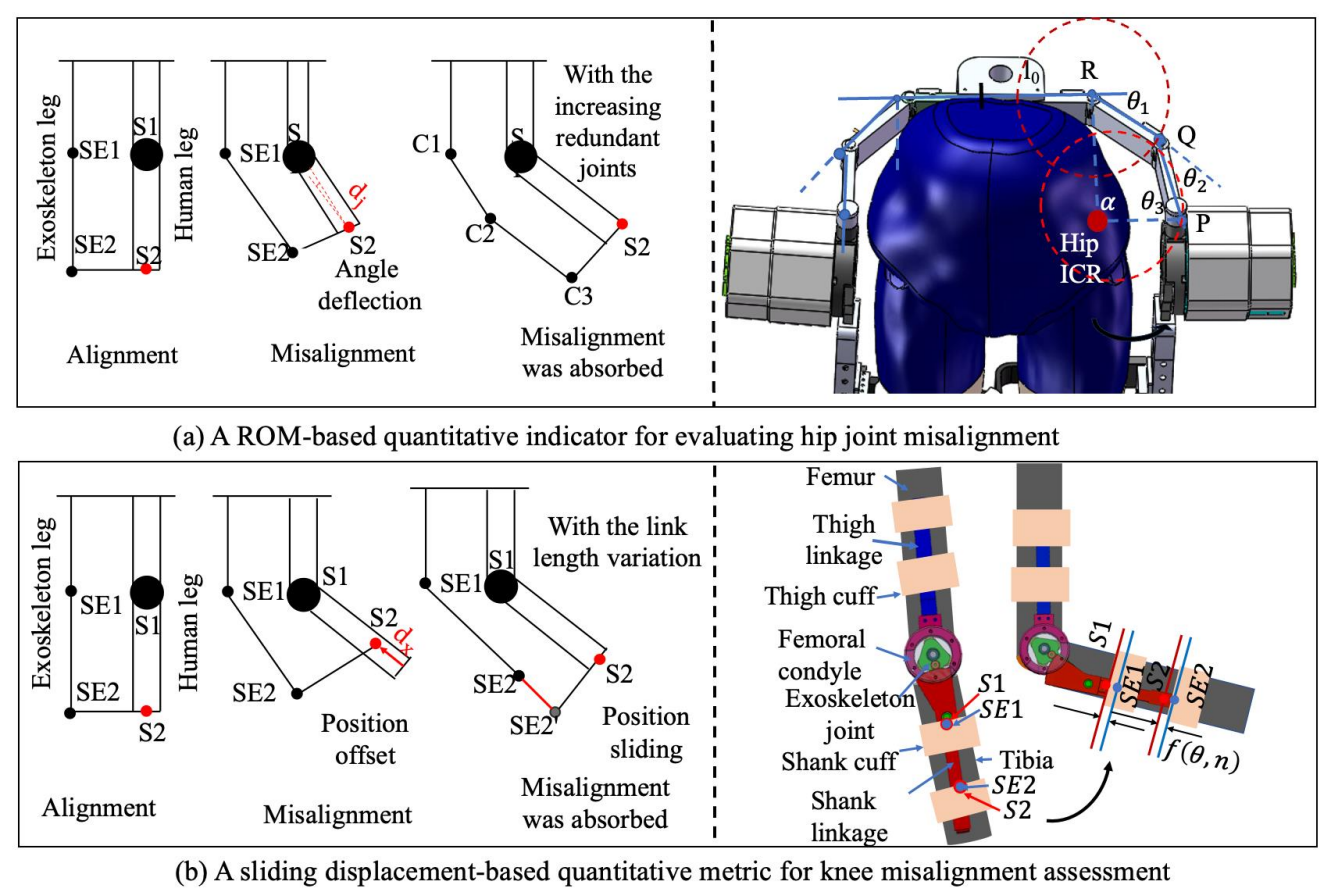
Two evaluation factors for misalignment evaluation.

To evaluate the biomimetic performance of the proposed exoskeleton design, this section proposes direct visualization of quantitative parameters of the human–exoskeleton contact interface to validate the kinematic misalignment resulting from the ICR inconsistency of human and exoskeleton. For the quantitative assessment of joint misalignment, two points on the exoskeleton, SE1 and SE2, are defined as corresponding to the two tracking markers, S1 and S2, affixed to the subject’s leg. Under ideal conditions of perfect alignment, points SE1 and SE2 would be coincident with markers S1 and S2, respectively. As depicted in Figure 7, when the human limb undergoes rotation, a 1-DOF mechanical joint results in a linear displacement, $d_{x}$, of the connection point C sliding along the human leg, or an angular displacement, $d_{j}$. Once the exoskeleton is permitted to alter the length of its links or to incorporate redundant joints, the connection point C remains unaffected. The rotational angles of the increasing joints or the modifications in link lengths could be construed as computational factors for misalignment.

#### 3.1.1. Hip Quantitative Evaluation and Results

The misalignment of hip joint resultant from pure rotational motion can be counteracted by the introduction of multiple additional rotational joints. This is exactly the method adopted in our design. As shown in Figure 7, the kinematic chain of the internal/external rotation composed of three revolute joints ($\theta_{1}$, $\theta_{2}$ and $\theta_{3}$) and three linkages ($L_{1}$, $L_{2}$, and $L_{3}$) conforms to the external profile of the human waist. It forms a four-bar mechanism OPQR with the human hip joint O, as depicted in Figure 2a. Upon considering the hip internal/external rotation angle α as the input angle, the equation governing the four-bar mechanism can be articulated:

(28) $$\left\{\begin{array}{c} \bar{OP}\sin\alpha +\bar{PQ}\sin\theta_{3}=\bar{QR}\sin\theta_{1}\\ \bar{OP}\cos\alpha +\bar{PQ}\cos\theta_{3}+\bar{QR}\cos\theta_{1}=\bar{OR} \end{array}\right..$$

It can be rewritten as:

(29) $$\left\{\begin{array}{c} \cos\theta_{1}=\left(\bar{OR}-\bar{OP}\cos\alpha -\bar{PQ}\cos\theta_{3}\right)/\bar{QR}\\ \sin\theta_{1}=\left(\bar{OP}\sin\alpha +\bar{PQ}\sin\theta_{3}\right)/\bar{QR} \end{array}\right..$$

By squaring both the equations of Equation (2) and adding them together, we can obtain a quadratic equation with respect to $cos\theta_{3}$. The solution can be represented as follows:

(30) cosθ2=ABD2±ABD22−B2−C2D212,

where

(31) $$A=2\bar{PQ}\left(\bar{OP}\cos\alpha -\bar{OR}\right),$$

(32) $$B={\bar{QR}}^{2}-{\bar{OR}}^{2}-{\bar{OP}}^{2}-{\bar{PQ}}^{2}+2\bar{OR}\bar{OP}\cos\alpha ,$$

(33) $$C=2\bar{PQ}\bar{OP}\sin\alpha ,$$

(34) $$D={\left(A^{2}+C^{2}\right)}^{1/2},$$

(35) $$\theta_{3}=\theta_{2}-\left(\pi -\alpha \right).$$

Then, $\theta_{3}$ and $\theta_{1}$ can be easily calculated:

(36) θ2=cos−1ABD2±ABD22−B2−C2D212,

(37) θ3=cos−1ABD2±ABD22−B2−C2D212+(π−α)

(38) $$\theta_{1}=\cos^{-1}\left(\bar{OR}-\bar{OP}\cos\alpha -\bar{PQ}\cos\theta_{4}\right)/\bar{QR}.$$

And

(39) $$\theta_{2}=2\pi -\theta_{3}-\theta_{1}-\alpha .$$

It follows that internal/external rotation angle of the exoskeleton is solely contingent upon the corresponding human hip internal/external rotation angle α. A misalignment compensation factor ∅ was defined as the discrepancy between the angular motion of the human hip and the exoskeleton hip. The factor ∅ is mathematically expressed as:

(40) $$\emptyset \left(\alpha \right)=\sqrt{{\left|\alpha_{exo}-\alpha_{h}\right|}^{2}},$$

where $\alpha_{exo}$ and $\alpha_{h}$ represent the actual exoskeleton rotation angle and the human angle, respectively.

**Figure 8:**
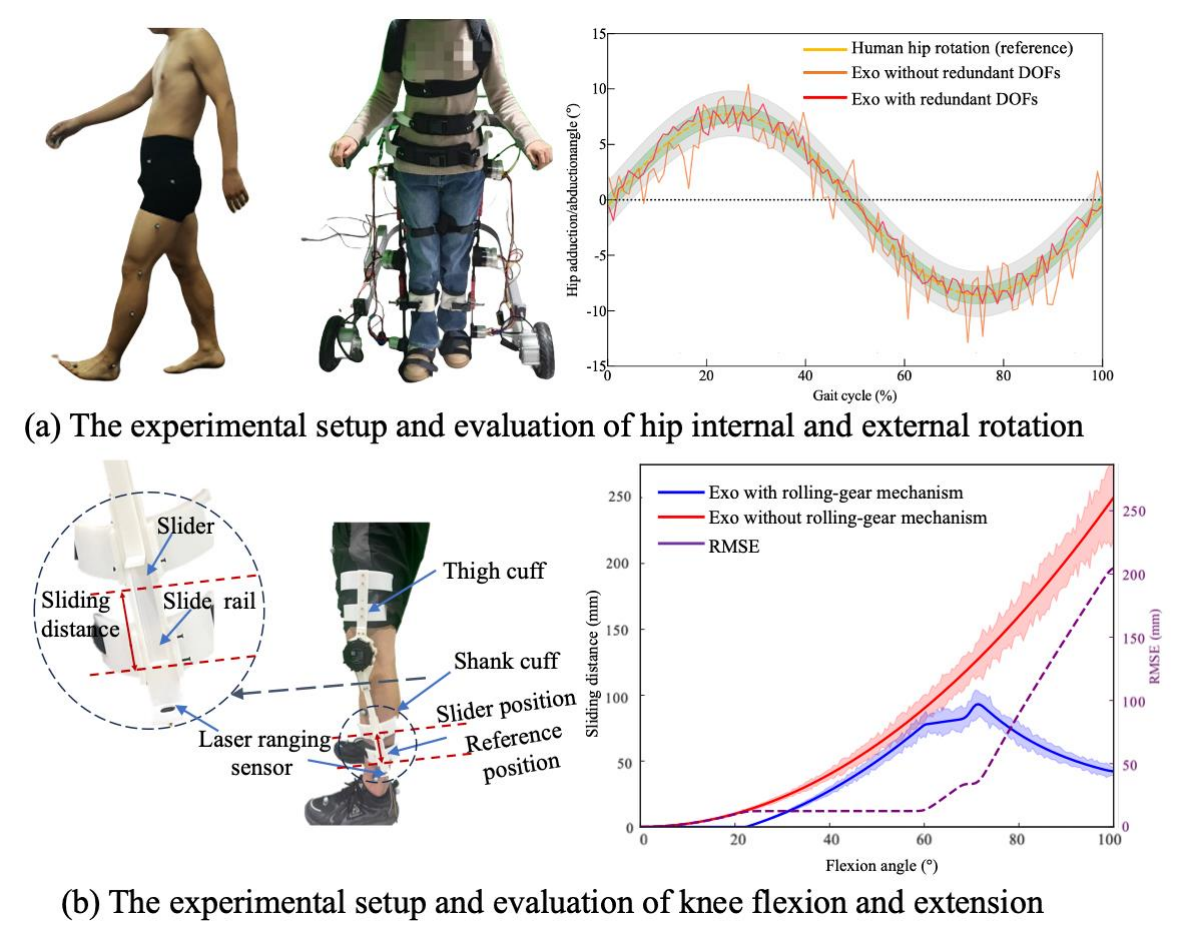
The experimental setup and evaluation results.

Figure 8a shows the comparative trajectories of hip rotation for both the human leg and the exoskeleton. The results demonstrate that with the redundant joint mechanism enabled, the exoskeleton closely followed the human hip rotation across the entire gait cycle. The supplementary joints introduced no observable mechanical interference. The mean RMSE decreased from 4.5°±1.2° (without compensation) to 2.3°±0.7° (with compensation). These findings demonstrate that the proposed self-aligning hip mechanism improves human–exoskeleton kinematic compatibility (66.8% reduction in misalignment) while preserving natural hip motion during walking.

#### 3.1.2. Knee Quantitative Evaluation and Results

To quantitatively characterize joint misalignment in the self-aligning design of the knee joint, misalignment attributable to the noncoincidence of motion trajectory is quantified as the sliding displacement along a translation direction perpendicular to the biological joint axis. This displacement can be captured using a sliding-connection mechanism, in which measured sliding distance is interpreted as the misalignment displacement. A custom-designed dual-slider measurement mechanism was implemented, as illustrated in Figure 8b. This system comprises the following two linear sliding elements: a slider rail integrated into the connection straps and a shank-mounted linear slider aligned with the exoskeleton frame. The sliders are mechanically coupled through custom 3D-printed components, which preserve structural alignment while permitting relative sliding motion.

During knee flexion and extension, the shank-mounted slider moves vertically along the limb, and the sliding displacement is continuously tracked. Notably, the sliding displacement is defined as the distance from the current position to a red-indicated reference point, as illustrated in Figure 8b. This displacement reflects the misalignment distance, providing a direct physical representation of ICR deviation between the biological and mechanical joints. The misalignment factor ∅ is defined as:

(41) $$\emptyset \left(\alpha \right)=\sqrt{\frac{1}{T}\int_{0}^{T}{\left|f\left(\theta \left(t\right),x\left(t\right)\right)\right|}^{2}dt},$$

where $f\left(\theta \left(t\right),x\left(t\right)\right)$ is the instantaneous sliding distance as a function of knee angle $\theta \left(t\right)$ and time-varying limb posture $x\left(t\right)$, and *T* is the gait cycle duration.

To measure this displacement in real-time, a laser-ranging sensor (MS53L0M) with a sampling rate of 160 Hz was mounted adjacent to the slider, enabling high-resolution distance tracking throughout the gait cycle. When employing the rolling joint mechanism, the misalignment error initially increases with the flexion angle. However, upon reaching a larger flexion angle (approximately 70°), the structure’s adaptive compensation mechanism becomes dominant, resulting in a downward trend in the error. This indicates that the proposed mechanism exhibits superior kinematic consistency and mechanical compliance in high-flexion regions. Compared with single-axis rotation joint (exoskeleton without the rolling-gear mechanism), the misalignment distance was reduced by approximately 87.4% at the maximum flexion angle typical of normal walking ($\theta_{max}=75{}^{\circ}$). These findings confirm the design’s effectiveness in improving kinematic alignment and ensuring motion congruence between human limb and exoskeleton.

### 3.2. Adaptability Evaluation of Abnormal Posture

**Figure 9:**
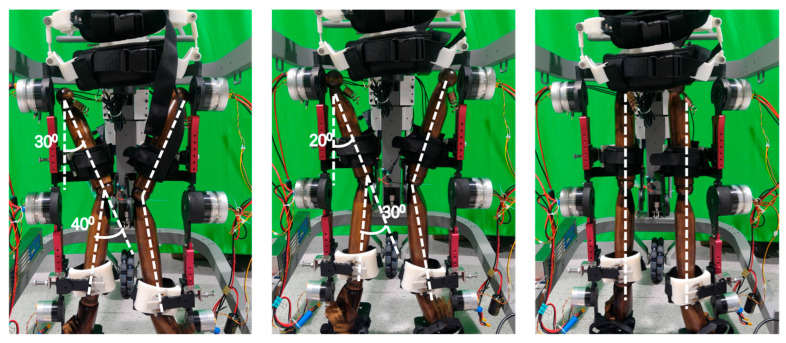
The child-sized anthropomorphic test platform.

To evaluate the exoskeleton’s capacity to accommodate abnormal lower-limb postures, a dedicated testing platform was developed to simulate various pathological gait features using a child-sized anthropomorphic test model. The model consists of two rigid links connected by a spherical joint, designed to replicate knee valgus deformity commonly observed in CP patients. During the experiment, different degrees of knee valgus were emulated by adjusting the angle between the two linked segments. The thigh and shank attachment straps of the exoskeleton were inclined at varying angles to assess the system’s ability to accommodate the changing joint configurations. The adaptation performance of the exoskeleton to varying knee joint angles is illustrated in Figure 9, which depicts the gradual correction from a valgus-aligned to a normal knee posture from left to right. The range of abnormal joint angles that the exoskeleton can accommodate is summarized in Table 2.

### 3.3. Evaluation of Unified Reference Phase Stability and Multi-Joint Coordination

A within-subject experimental design was adopted, with each participant tested under the following control conditions in randomized order: (1) baseline: the exoskeleton was worn with minimal assistance, providing only gravity compensation and passive support, serving as a reference for natural gait; (2) proposed unified phase control: the proposed method employing multi-joint local gait phase estimation, adaptive phase fusion to construct a unified reference phase, and phase-consistency-based joint coupling.

Participants performed over-ground walking along a straight walkway at a self-selected comfortable speed. The proposed control framework was implemented in real time on an STM32-based embedded controller. The average computation latency per control cycle was approximately 1–2 ms, which is well below the control sampling period (10 ms, corresponding to 100 Hz). During experimental operation, CPU utilization remained below 30%, ensuring stable real-time execution throughout all trials.

Lower-limb joint angles were recorded using a Vicon Motion Capture System (Oxford Metrics, UK) with a sampling frequency of 160 Hz. Each trial lasted 60 s, during which at least eight consecutive gait cycles were analyzed. Gait parameters, including step length, cadence, and stride time, were also extracted to quantify temporal–spatial gait characteristics.

#### 3.3.1. Phase Consistency and Multi-Joint Coordination

**Figure 10:**
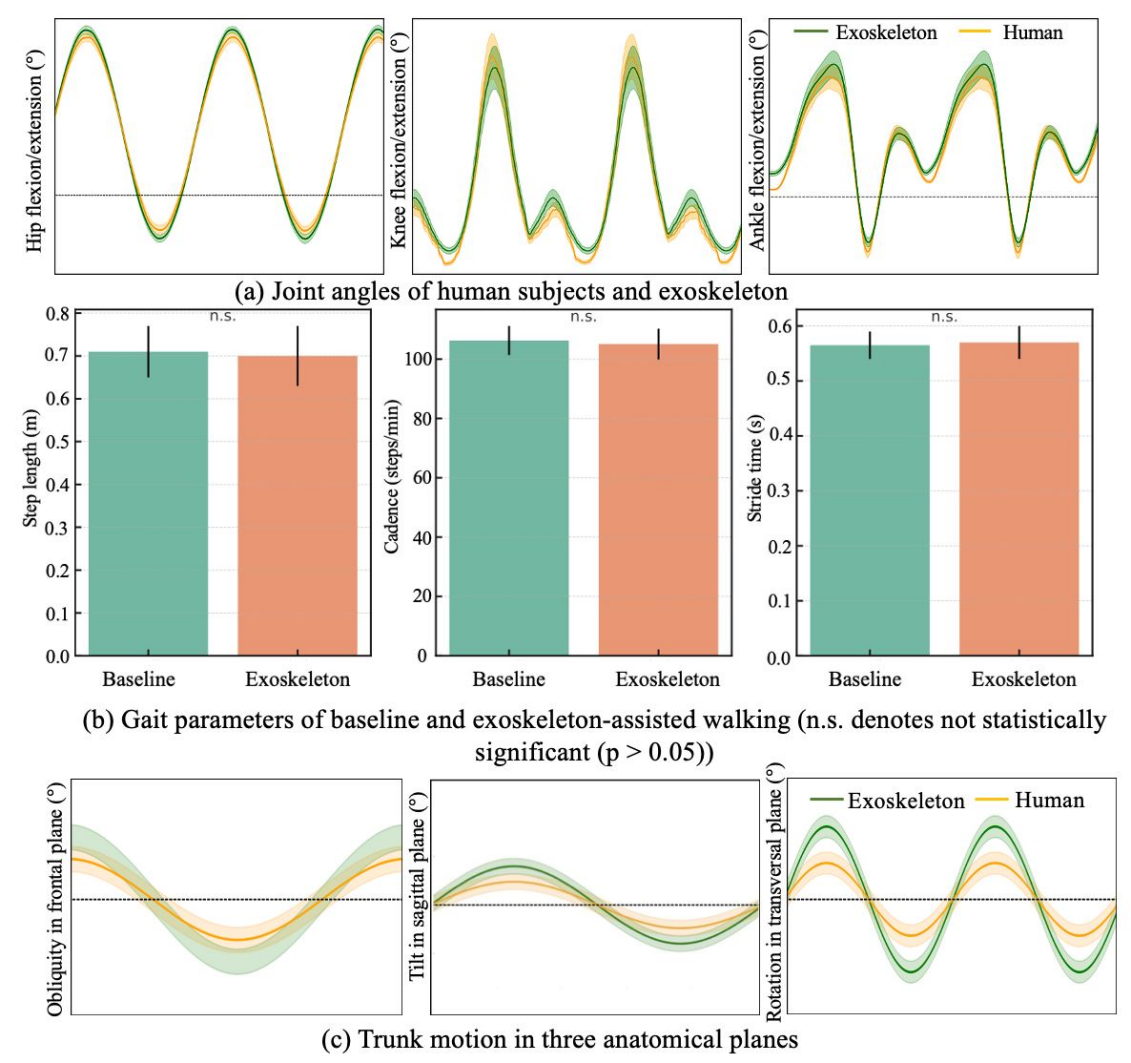
Motion parameters of human–exoskeleton walking.

As shown in Figure 10a, the hip, knee, and ankle joint trajectories exhibited consistent and smooth evolution over the normalized gait cycle under the proposed unified phase control. Across all joints, the mean trajectories followed physiologically typical flexion–extension patterns without abrupt discontinuities or phase distortions, indicating stable progression of the underlying gait phase reference.

Importantly, the timing of key kinematic events was well-aligned across joints. The hip flexion–extension trajectory demonstrated a clear and repeatable transition between stance-related extension and swing-related flexion, which coincided with the onset of knee flexion and the subsequent ankle plantarflexion during push-off. Similarly, the peak knee flexion during swing occurred in synchrony with hip flexion and ankle dorsiflexion, reflecting preserved inter-joint timing relationships throughout the gait cycle.

The relative variability bands observed across all three joints further indicate the unified gait phase reference provided a stable and variable temporal framework shared by multiple joints. Rather than each joint evolving according to an independent local phase, the proposed approach effectively coordinated hip, knee, and ankle motions within a common phase progression, thereby maintaining coherent multi-joint kinematic structure.

This phase consistency information is used to modulate phase-dependent control parameters rather than enforcing strict joint-level phase locking. Specifically, when inter-joint phase consistency is high, the controller applies more concentrated reference tracking, resulting in tighter trajectory convergence. When phase consistency decreases, control parameters are adaptively adjusted to relax trajectory constraints, allowing joint motions to vary within a bounded range while maintaining alignment with the unified gait phase.

#### 3.3.2. Spatiotemporal Gait Parameters

Figure 10b displays key spatiotemporal gait parameters, including step length, cadence, and stride time under baseline and exoskeleton-assisted conditions. The average step length was 0.71±0.06 m during baseline walking and 0.70±0.07 m with exoskeleton assistance. Cadence showed a comparable trend, with values of 106.3±4.9 steps/min and 105.1±5.2 steps/min for the baseline and assisted conditions, respectively. Similarly, the mean stride time remained nearly unchanged (1.13±0.05 vs. 1.14±0.06).

Overall, no statistically significant differences were observed in any of the spatiotemporal parameters between the two conditions (p>0.05), indicating that the proposed control strategy preserved the natural temporal and spatial characteristics of gait.

### 3.4. Torso Kinematics Evaluation of No Interference by Biofeedback Strategy

To evaluate the adaptability of the trunk exoskeleton in accommodating human postural deviations and ensure conformity with the kinematic demands of torso motion during exoskeleton-assisted walking, a real-time biofeedback strategy based on inertial sensing was adopted as the principal method in this validation. Here, the term “biofeedback” refers to an IMU-based acoustic cue for user awareness rather than any form of active robotic posture control.

Two inertial measurement units (IMUs; sampling rate 100 Hz, 9-axis) were used to monitor trunk orientation—one affixed to the participant’s forehead and the other to the upper sternum. During walking with the exoskeleton, if the trunk angle exceeded the preset limits in any plane, an acoustic signal was triggered to prompt immediate self-correction. The exoskeleton did not actively enforce posture correction but passively supported the trunk, relying on the participant’s voluntary adjustment in response to sensory feedback.

Prior to data collection, participants were given sufficient familiarization time with the exoskeleton and the acoustic feedback cues to minimize transient adaptation effects. Because BWS module is mechanically coupled to the trunk exoskeleton in the proposed system, it was included in this experiment as part of the trunk support structure. This configuration reflects the actual operating condition when evaluating whether the trunk exoskeleton constrains abnormal motion within a physiological range without interfering with normal trunk activity.

Figure 10c illustrates the trunk angle trajectories across the sagittal, frontal, and transverse planes during exoskeleton-assisted ambulation. In all three planes, the actual trajectories (orange curves) generally followed the shape of the reference curves (green curves), indicating that participants were able to voluntarily adjust their posture in response to acoustic feedback when deviations exceeded preset thresholds. Notably, in frontal plane, the actual curve closely tracked the reference with minimal phase lag, showing effective lateral balance during gait. In sagittal plane, a slight amplitude reduction was observed, suggesting that participants maintained a slightly more upright posture with the exoskeleton, potentially due to its passive trunk support. In transverse plane, despite some deviation, the phase and general trend remained consistent with the reference, demonstrating that axial trunk rotation was preserved. Taken overall, the exoskeleton did not restrict natural torso motion.

## 4. Discussion

### 4.1. Trunk–Limb Integrated Design for Pediatric CP Gait Rehabilitation

The present study addresses the following critical but underexplored aspect of pediatric CP gait rehabilitation: the tight coupling between trunk control and lower-limb coordination during walking. Unlike most existing pediatric exoskeletons that primarily focus on sagittal-plane lower-limb assistance, the proposed system explicitly integrates a trunk support module with a self-aligning lower-limb exoskeleton. By incorporating trunk stabilization as a physical constraint rather than a purely sensory or cognitive cue, the proposed system targets gait correction at the whole-body level.

In the present study, the trunk module is primarily intended as a passive postural stabilization and guidance interface. Its main role is to provide configurable mechanical constraints that limit excessive trunk deviations while preserving physiological trunk motion during walking. By offering scalable support through adjustable height and width configuration, the trunk module establishes a stable postural reference for lower-limb coordination without directly imposing corrective torques. In this sense, the module functions both as a passive stabilizing structure and as a preparatory platform that can accommodate future extensions toward active trunk assistance.

### 4.2. Effectiveness of Self-Aligning Mechanisms in Enhancing Human–Machine Compatibility

Human–exoskeleton misalignment remains a major source of discomfort, parasitic forces, and motion interference, especially in pediatric users with heterogeneous anthropometry and pathological gait patterns. Compared with single-axis rotation joint, the proposed hip self-alignment mechanism achieved a 66.8% reduction in rotational misalignment using a compact redundant revolute-chain design, while the gear-based rolling knee joint reduced sliding misalignment by 87.4% at physiologically relevant flexion angles without relying on large translational joints or remote centers.

This combination of high misalignment reduction and relatively simple mechanical architecture distinguishes the proposed design from existing solutions, offering a practical balance between kinematic compatibility, added mass, and wearable complexity—an important consideration for pediatric exoskeleton applications.

### 4.3. Unified Gait Phase Control Under Pathological Gait Variability

From a control perspective, one of the key challenges in CP exoskeleton assistance lies in handling irregular inter-joint timing, asymmetric motion, and stride-to-stride variability. Conventional gait phase estimation approaches based on discrete finite-state machines or single-joint timing often struggle under these conditions, potentially leading to mistimed or intrusive assistance.

The proposed continuous gait progress-based control framework provides a fundamentally different solution by representing gait timing as a circular, continuous variable and fusing multi-joint phase estimates into a unified system-level reference. By weighting each joint’s contribution according to its instantaneous observability, the method remains robust when individual joints exhibit spasticity, reduced excursion, or unreliable kinematic signals. This is particularly advantageous for CP gait, where no single joint can be assumed to provide a consistently reliable timing reference.

Moreover, inter-joint coordination is achieved through phase-consistency-based temporal modulation rather than direct kinematic enforcement. By adjusting the rate of phase evolution instead of imposing strict trajectory constraints, the controller preserves joint-level autonomy and allows bounded variability in joint motion. The observed preservation of spatiotemporal gait parameters under assisted conditions supports the notion that the proposed strategy provides transparent assistance that does not override the user’s intrinsic gait rhythm.

Although nominal joint trajectories derived from calibration data are used as reference templates for gait progress estimation, the proposed unified gait phase control strategy is not based on strict trajectory tracking and does not assume close adherence to nominal kinematic patterns. Robustness to large deviations—common in pathological CP gait—is achieved through independent joint-level phase estimation and observability-weighted phase fusion, which adaptively reduces the influence of joints exhibiting atypical or unreliable motion. As a result, deviations from nominal patterns primarily affect confidence weighting rather than the validity of the unified gait phase, thereby reducing sensitivity to pathological variability and limiting the need for frequent recalibration. In clinical settings, nominal trajectories can be obtained during an initial fitting session and reused across sessions, with recalibration required only when substantial and persistent changes in gait patterns occur.

### 4.4. Limitations and Future Directions

Several limitations of the present study should be acknowledged. First, the technical validation in this study was conducted on healthy adult participants. This approach is appropriate for initial technical validation, as it allows the assessment of engineering feasibility, including mechanical compatibility, control stability, and transparency of assistance, under well-controlled and repeatable gait conditions. The present results should be interpreted as evidence of engineering feasibility rather than demonstrated clinical efficacy. It is acknowledged that the primary clinical challenge for pediatric CP exoskeletons lies in accommodating a highly variable and unstable pathological gait rather than regular walking patterns. Accordingly, while the present results establish the mechanical feasibility and control robustness of the proposed trunk–limb exoskeleton, future clinical studies will focus on assessing system robustness under pathological gait variability and evaluating functional outcomes through longer-term use in children with cerebral palsy.

Second, trunk assistance in the current implementation is passive, relying on structural support and biofeedback rather than active actuation. Future work will extend the current passive trunk support toward active trunk assistance to enable more flexible and adaptive control. In particular, active trunk exoskeleton designs based on linear actuators may be explored to provide controllable postural assistance, allowing trunk support to be modulated according to user capability, gait phase, or stability demands. Such active trunk assistance has the potential to complement the existing adjustable structure and further enhance adaptability for users with severe trunk control impairments.

Additionally, while the unified phase framework effectively coordinates lower-limb joints, integration with higher-level intent detection or therapist-defined rehabilitation goals remains an open research direction. Incorporating learning-based adaptation or patient-specific progression of assistance could further enhance clinical outcomes.

## 5. Conclusions

This study presented a novel trunk–limb exoskeleton system designed to support gait rehabilitation in children with CP and related neuromotor disorders. The system combines a trunk module for postural stabilization with a lower-limb module for active gait assistance, thereby enabling functional ambulation in overground environments. Technical evaluations confirmed that the device provides effective trunk support without restricting lower-limb mobility.

Beyond mechanical integration, a continuous gait progress-based multi-joint control framework was developed to address the abnormal inter-joint timing and variability characteristic of pediatric CP gait. By representing joint motion as continuous gait progress over the full gait cycle and unifying multi-joint timing using observability-weighted circular statistics, the proposed control method establishes a robust system-level temporal reference without relying on discrete gait phase classification. Phase-consistency-based temporal modulation implemented via velocity feedforward enables smooth inter-joint synchronization while maintaining joint-level autonomy and transparency.

In contrast to existing exoskeletons that primarily focus on isolated lower-limb assistance, the proposed system emphasizes trunk and lower-limb cooperative motion and temporally coordinated assistance, enhancing postural stability, kinematic compatibility, and user comfort. The inclusion of self-alignment mechanisms further reduces joint misalignment and compensatory motion, while the pediatric-specific design accommodates children’s anatomical characteristics and gait patterns. Future work will focus on clinical validation in children with CP to evaluate long-term rehabilitation outcomes and therapeutic efficacy.

## Figures and Tables

**Table 1:** The parameters of gears.

Gear	Module	Number of Teeth
Sun Gear	1.0	48
Planet Gear	1.0	30
Ring Gear	1.0	108
Gear 1	1.0	24
Gear 2	1.0	28
Gear 3	1.0	120

**Table 2:** Adaptation range of exoskeleton to abnormal posture.

Joint Motion	Range of Motion
Hip adduction	0°–30°
Hip flexion	0°–90°
Knee valgus	0°–40°
Knee flexion	0°–90°

## Data Availability

The data are not publicly available due to privacy or ethical restrictions.

## Abbreviations

The following abbreviations are used in this manuscript:

CP	Cerebral palsy
DOF	Degree of freedom
BWS	Body-weight support
